# Comparing Public Sentiment Toward COVID-19 Vaccines Across Canadian Cities: Analysis of Comments on Reddit

**DOI:** 10.2196/32685

**Published:** 2021-09-24

**Authors:** Cathy Yan, Melanie Law, Stephanie Nguyen, Janelle Cheung, Jude Kong

**Affiliations:** 1 Department of Genome Science and Technology University of British Columbia Vancouver, BC Canada; 2 Department of Microbiology and Immunology University of British Columbia Vancouver, BC Canada; 3 Department of Biomedical Engineering University of British Columbia Vancouver, BC Canada; 4 Department of Biochemistry University of British Columbia Vancouver, BC Canada; 5 Department of Mathematics & Statistics York University Toronto, ON Canada

**Keywords:** COVID-19, public sentiment, social media, Reddit, Canada, communication, sentiment, opinion, emotion, concern, pandemic, vaccine, hesitancy

## Abstract

**Background:**

Social media enables the rapid consumption of news related to COVID-19 and serves as a platform for discussions. Its richness in text-based data in the form of posts and comments allows researchers to identify popular topics and assess public sentiment. Nonetheless, the vast majority of topic extraction and sentiment analysis based on social media is performed on the platform or country level and does not account for local culture and policies.

**Objective:**

The aim of this study is to use location-based subreddits on Reddit to study city-level variations in sentiments toward vaccine-related topics.

**Methods:**

Comments on posts providing regular updates on COVID-19 statistics in the Vancouver (r/vancouver, n=49,291), Toronto (r/toronto, n=20,764), and Calgary (r/calgary, n=21,277) subreddits between July 13, 2020, and June 14, 2021, were extracted. Latent Dirichlet allocation was used to identify frequently discussed topics. Sentiment (joy, sadness, fear, and anger) scores were assigned to comments through random forest regression.

**Results:**

The number of comments on the 250 posts from the Vancouver subreddit positively correlated with the number of new daily COVID-19 cases in British Columbia (*R*=0.51, 95% CI for slope 0.18-0.29; *P*<.001). From the comments, 13 topics were identified. Two were related to vaccines, 1 regarding vaccine uptake and the other about vaccine supply. The levels of discussion for both topics were linked to the total number of vaccines administered (Granger test for causality, *P*<.001). Comments pertaining to either topic displayed higher scores for joy than for other topics (*P*<.001). Calgary and Toronto also discussed vaccine uptake. Sentiment scores for this topic differed across the 3 cities (*P*<.001).

**Conclusions:**

Our work demonstrates that data from city-specific subreddits can be used to better understand concerns and sentiments around COVID-19 vaccines at the local level. This can potentially lead to more targeted and publicly acceptable policies based on content on social media.

## Introduction

Sixty-five percent of approximately 3.8 billion internet users are currently informed about top news stories from social media platforms such as Twitter, Facebook, and Reddit rather than traditional news outlets [1]. Surveys show that this trend has been especially apparent during the COVID-19 pandemic as more people seek timely updates on the crisis [2]. When used effectively, social media platforms can disseminate relevant health-related information to users such as patients, clinicians, and scientists [1]. However, the unfamiliarity of COVID-19 has led to the frequent transmission of false and conflicting information [3]. While awareness of “fake news” is high among Gen Z individuals and Millennials, less than a quarter of them report posts with false information, and only 8.7% opt to stop receiving updates from the account that produces misleading posts [4]. Propagated misinformation also negatively impacts compliance with public health policies such as social distancing [5].

In addition to what people express on social media, investigation of their underlying attitudes in conjunction with their comments can be key to determine political participation and predict “protester violence” [6,7]. In the context of the pandemic, posts on Twitter and Facebook have been used to examine attitudes toward contact tracing apps in the United Kingdom [8], and a dashboard was built to track emotions in Austria on the basis of the news platform derstandard.at, Twitter, and a chat platform for students [9].

Currently, one of the most critical steps to reducing the spread of COVID-19 is mass vaccination [10]. Unfortunately, social media also provides a platform for growing antivaccination movements and increasing vaccine hesitancy [11-13]. When examining the expression of these opinions that oppose scientific advice, studies capture sentiments across entire platforms or whole countries but fail to capture nuances at a more local level. Thus, we sought to use comments on Reddit to explore discussions surrounding COVID-19 in Toronto (Ontario), Calgary (Alberta), and Vancouver (British Columbia) as sentiment analysis can contribute to improving social management practices within each city.

Reddit is divided into subreddits, which contain posts and discussions relevant to a particular location or topic. As a platform that quickly aggregates content, it effectively disseminates the latest news regarding major events, including the COVID-19 pandemic. For example, daily posts on the Vancouver subreddit (r/vancouver), by the user cyclinginvancouver (u/cyclinginvancouver), provide updated statistics regarding the spread of COVID-19 in British Columbia. Similar posts can be found on the Calgary (r/calgary) and Toronto (r/toronto) subreddits. The posts themselves are unbiased, containing only information such as the number of new cases of COVID-19 infection as determined by a positive test, hospitalizations, vaccinations, and deaths. Thus, people are able to engage in free-form discussion in the comments. Comparatively, other platforms such as Twitter allows a global community to discuss the pandemic; hence, discussions are less specific to regional communities.

The aim of this study is to use location-based subreddits on Reddit to study city-level variations in sentiments toward COVID-19 vaccine–related topics. Here, we first present an analysis of comments from the Vancouver subreddit to understand the topics being discussed and people’s attitudes toward local policies. We also specifically explore people's reactions and sentiments toward vaccines and how they align with vaccination rates. Then, we characterize differences in vaccine topics and sentiments among Vancouver, Toronto, and Calgary. Data from similar studies performed using Twitter [14] helped build some of our models, as our study is the first to examine comments from Reddit in this manner. Our novel approach of examining topics of local concern have the potential to inform how public policy can be tailored to specific geographical regions.

## Methods

### Data Collection

All analyses were performed using Python (version 3.7) and R (version 4.0.2). Our code can be found on GitHub at Mellaw/BDC_Reddit.

Reddit comments were acquired using the Python Reddit API Wrapper (PRAW; version 7.2.0) [15]. A read-only Reddit instance was created and used to obtain a subreddit instance for the Vancouver, Calgary, and Toronto subreddits (r/vancouver, r/calgary, and r/toronto, respectively). Relevant posts were acquired by searching the keywords *Covid-19 Update -*, *Alberta Totals:*, and *COVID-19 in Ontario* in the Vancouver, Calgary, and Toronto subreddits, respectively. Keywords were selected to be specific to posts providing daily updates on COVID-19 statistics. The title, time created, submission ID, and author were extracted and assembled into a data frame using pandas (version 1.2.4) [16]. The submission ID is a string unique to each post. It was used as an input to PRAW for interacting with each post’s “CommentForest” or a list of comments and replies. The list method of CommentForest was used to extract the body text, author, and title of the post for all comments.

Twitter data were obtained from the paper “Global Reactions to COVID-19 on Twitter: A Labelled Dataset with Latent Topic, Sentiment and Emotion Attributes” [14] and were made available on OpenICPSR [17]. We used the version with 5000 tweets randomly sampled from the full data set of 132.1 million tweets. The tweets are in English and are from the United States, Singapore, India, and Brazil. Every tweet was scored on how intensely they demonstrated the emotions anger, fear, sadness, and joy on a scale from 0 to 1. Since the data set only provided the IDs of tweets, we used Tweepy (version 3.10.0) to hydrate the tweets [18].

We also used statistics for new cases of COVID-19 and total vaccinations in British Columbia, Alberta, and Ontario. These data, as well as numbers for fatalities, hospitalizations, tests, and recoveries, were provided through a web-based COVID-19 Tracker [19], where real-time data at both the national and provincial level were collected by volunteers. To ensure accuracy, statistics were updated primarily from live press briefings, with some supplementation from news networks. Source URLs were also provided.

### Data Processing

#### Preprocessing Raw Text

All Reddit comments and Tweets were converted to lowercase, and the Python software package [20] was used to remove nonalphabetical characters, URLs, and references to other users. Texts were then lemmatized using spaCy (version 3.0) [21] and stripped of stop words using Natural Language Toolkit (NLTK; version 3.6.2) [22]. The list of stop words was modified by removing “no” and “not.”

#### Topic Extraction

Topic extraction was performed for Reddit comments by implementing Latent Dirichlet Allocation (LDA) through gensim (version 4.0.1) [23]. After tokenizing, comments were used to create a dictionary, which maps every word to a unique integer ID. This was then converted into a bag-of-words format, essentially counting how many times a word is used. To optimize the number of topics, LDA models were created for 1 to 14 topics. Other nondefault parameters include setting the chunk size to be the number of comments, the number of passes to 10, and α to “auto.”

The optimal number of topics was deemed to be the one that minimizes mean Jaccard similarity while maximizing mean coherence across topics. Jaccard similarity is a metric for how many words 2 documents have in common [24]. If 2 topics were identical, their Jaccard similarity would be 1. Conversely, if 2 topics shared no words in common and were thus entirely distinct, their Jaccard similarity would be 0. Coherence for each topic was calculated with gensim using the “c_v” option. It measures the semantic similarity between high-scoring words, which is a function of how often the words co-occur across comments [25].

Each comment was quantitatively assessed to what extent they addressed each topic with a score from 0 to 1. For downstream analyses, a comment was considered to address a topic (“1”) if the score was greater than 0.2 and not (“0”) otherwise. Word clouds showing the top keywords for each topic were generated using wordcloud (version 1.8.1) and Matplotlib (version 3.4.2). Thus, we were able to identify vaccine-related comments.

#### Sentiment Analysis

Scores for emotional intensity were assigned to Reddit comments using a model built from the Twitter data set. Using scikit-learn (version 0.24), the Twitter data set was split into training (0.75) and testing (0.25) sets. Prior to modeling, the text of the tweets was processed into a data frame containing term frequency–inverse document frequencies (TF–IDF). The term frequency (TF) is the number of times a word appears in each tweet divided by the total number of words in that tweet. The inverse document frequency (IDF) is the logarithm of the total number of tweets divided by the number of tweets containing the word. TF–IDF is simply the TF multiplied by the IDF.

The TF–IDF data frame was used as the input to a random forest regression model. The model from scikit-learn was used with default parameters. The scores for anger, fear, sadness, and joy were the target variables. A total of 4 models were fitted, 1 for each emotion. To evaluate the models, the data in the TF–IDF data frame for the test data set were used as the predictors, and the root mean square error (RMSE) was used to compare model outputs with true values. The same processing workflow and models were applied to Reddit comments to predict emotional intensity.

#### Statistics

The correlation between the number of Reddit comments and new cases of COVID-19 was calculated using Pearson correlation in R. Time-series trends for the level of vaccine-related discussion and total vaccinations in British Columbia were compared using the Granger causality test from the lmtest package [26]. Comparisons across topics and cities were performed using the Mann–Whitney *U* test.

## Results

### Data Overview

From the r/vancouver subreddit, 49,291 comments across 250 daily update posts were obtained between July 13, 2020, and June 14, 2021. After preprocessing, 433 comments were found to be duplicated. To avoid including spam, duplicates from technical glitches, and placeholder text for deleted and removed comments, duplicates were excluded from further analysis, yielding 45,303 usable comments. These comments were contributed by 4261 users (“Redditors”). Summary statistics for r/calgary and r/toronto are also displayed in Table 1.

**Table 1:** Summary statistics for posts and comments extracted.

City	Total posts, n	Total comments, n	Usable comments, n
Vancouver	49,291	250	45,303
Toronto	20,764	234	19,105
Calgary	21,277	249	18,886

### Vancouver-Specific Analyses

#### Engagement Level on Reddit Correlates With Daily New COVID-19 Cases

**Figure 1:**
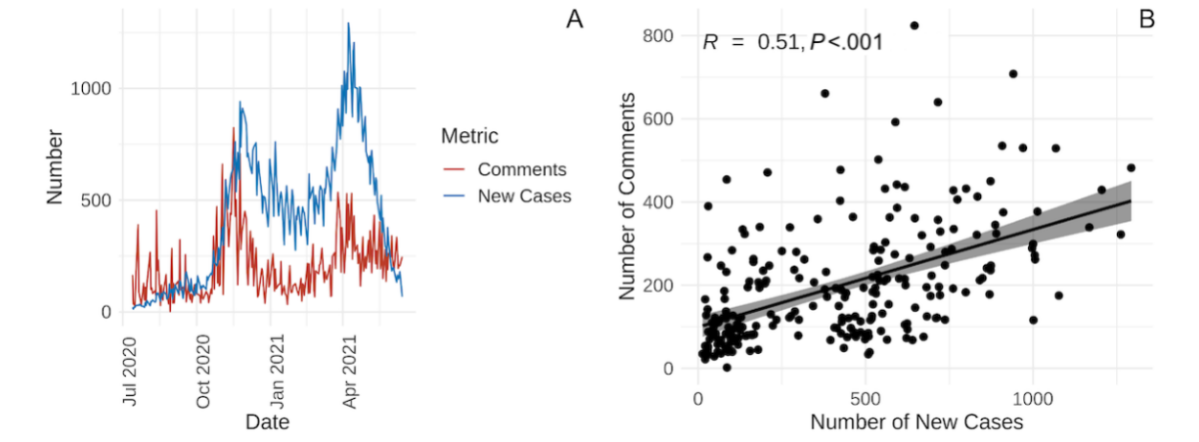
(A) Line plot depicting the number of new COVID-19 cases in British Columbia (blue) and the number of comments on each daily update post (red) from July 13, 2020, to June 14, 2021. (B) The number of new COVID-19 cases is significantly positively correlated with the number of comments on daily update posts (*R*=0.51; *P*<.001).

The number of new COVID-19 cases in British Columbia has 2 distinct peaks (Figure 1A). The first is in late November 2020, following a period of low, steady numbers in the summer, with exponential growth beginning in October 2020. The second is in early April 2021, after a local minimum in February 2021, and preceding a steep decline. The number of comments on daily update posts demonstrates a similar trend over time and was found to be significantly correlated with the number of new COVID-19 cases (*R*=0.51, 95% CI for slope 0.18-0.29; *P*<.001) (Figure 1B).

#### Thirteen Main Topics Related to COVID-19 Were Identified From Reddit Comments

By maximizing coherence and minimizing Jaccard similarity, the ideal number of topics was deemed to be 13 (Multimedia Appendix 1). Based on the word clouds (Multimedia Appendix 2) and examples of the first 25 words for the highest scoring comment for each topic (Table 2), the topics are as follows: (1) advocating for restrictions, (2) COVID-19 transmission, (3) impacts of COVID-19 on social spheres, (4) discussion about case numbers, (5) outbreaks in health care facilities, (6) debating how realistic public health orders are, (7) scientific concepts surrounding COVID-19, (8) monitoring travelers and people who have been exposed, (9) violating and enforcing restrictions, (10) vaccine uptake, (11) general speculations, (12) impact on hospitals, and (13) vaccine scarcity.

**Table 2:** Examples of processed comments with the highest score for each topic.

Topic	Processed comment
1	“well nt make zero case still strong restriction truck driver example implement policy nt truck unload instead local worker local worker also test daily one”
2	“not interpretation public health monitoring figure individual direct self isolate day know exposure identify contact tracing question yesterday student return class leave self isolation day”
3	“problem collective reason spread happen exponentially community strong social tie custom obligation ie wedding season lot young adult ca nt live together prior marriage taboo parent”
4	“monday multi day count new case consistent trend flat growth plateaue no acceleration no deceleration seven day trail average basically unchanged per day value near”
5	“six new healthcare facility outbreak braddan private hospital kin village madison care centre royal city manor william lake senior village creekside land outbreak chilliwack“
6	“isolation not fast literally never eliminate virus not canada certainly not globally country temporarily locally eliminate it which coincidentally island nation even island invariably virus introduce”
7	“vaccine currently use new mrna base one reprogram surface marker protein make machinary cell make virus spike protein immune system detect build resistance virus”
8	“people active public health monitoring result identify exposure know case active case recover case vancouver coastal health region case fraser health region case interior health region”
9	“nt see enforcement feasible transit security every bus take last night home bus driver keep tell people bus full pull ahead stop rather let people”
10	“reassurance calculation bc receive vaccine dose percentage first dose adult population date thursday usually shipment dose cumulative march dose march dose march dose april st dose”
11	“selectively ignore scientist scientist science deem trustworthy work right we medium company not part pov actually know people like answer question base see yes no”
12	“nine hospital bc vancouver coastal fraser health move emergency surgery least next two week mean combine elective surgery cancel low mainland fraser health abbotsford burnaby surrey memorial”
13	“parent age range little hesitant well reason azd vaccine cautious approach sure pfizer moderna slightly high effectiveness rating age group still month month half away”

#### Increase in Vaccine-Related Discussion Correlates With the Number of People Vaccinated

**Figure 2:**
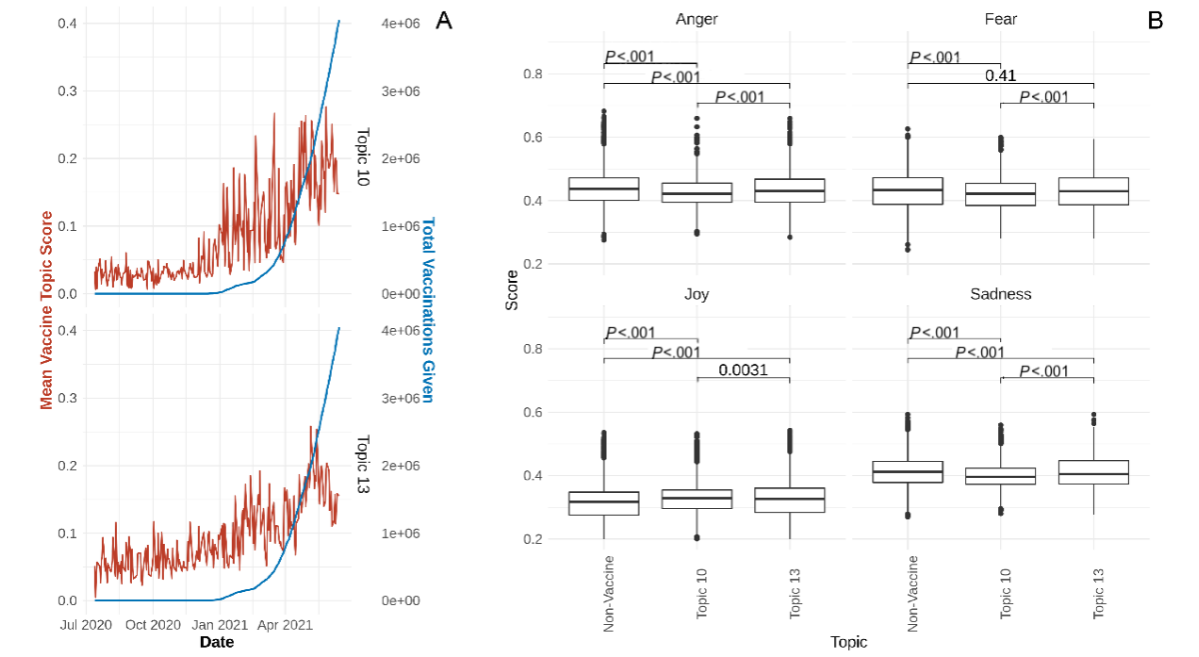
(A) Line plot displaying the daily average vaccine topic score (red) for topics 10 (top) and 13 (bottom) and the total number of vaccines administered up to that date (blue). (B) Box plots showing the distribution of emotional intensity scores. Comparisons between groups were made using the Mann–Whitney *U* test.

Specifically focusing on discussions surrounding vaccines, we see that the topic scores for topics 10 and 13 began to trend upward starting in January 2021, with topic 13 being slightly more highly discussed before then (Figure 2A). When overlaid with data on the total number of people vaccinated in British Columbia [19], we see that the rise in discussion precedes the rise in the number of vaccinations, although both trend similarly. The Granger test for causality was performed to evaluate the impact of vaccine discussion on total vaccinations. The results were significant for both topics (*P*<.001).

#### Vaccine-Related Comments Express Significantly Higher Positive Sentiments

Random forest regression models built using tweets labeled with emotional intensity scores were evaluated using RMSE values (Multimedia Appendix 3). Even when applied to Reddit comments, the model appears to retain validity. For each emotion, the processed comment with the highest score is displayed in Table 3. Additionally, the negative emotions (sadness, fear, and anger) are significantly, strongly positively correlated with each other and negatively correlated with joy (Multimedia Appendix 3).

**Table 3:** Examples of processed comments with the highest intensity scores for their respective emotions.

Emotion	Score	Processed comment
Joy	0.54	“depend happy plateau hit case day long time okay case day stay steady thing look cautiously optimistic definitely call”
Sadness	0.59	“not scientific datum notice friend indocanadian community really enthusiastic vaccine due really sad outlook india covid situation right lot family back india truly suffer take quite seriously”
Anger	0.68	“fucking sick people not give fuck people understand pass around hospital shit show soon”
Fear	0.63	“care home care home interior home town announce outbreak today mom nurse different care home town worried transmission partner lose grandparent already quarantine non covid today two fear come true sick scare sorry”

From July 2020 to April 2021, the emotional intensity scores were steady for all emotions (Multimedia Appendix 3). Negative emotions were expressed more than joy, with anger being the most prominent. From April 2021 onward, the mean scores for joy begin to trend upward while those for negative emotions all trend downward. Investigating the differences in emotional intensity scores between vaccine-related and non–vaccine-related comments, we found that comments for both vaccine topics had significantly higher scores for joy (*P*<.001) (Figure 2B). However, comments regarding vaccine availability had significantly lower scores for joy and higher scores for negative emotions (*P*<.001) compared to comments about vaccine uptake.

### Comparison Across Cities

#### Stronger Positive-Sentiment is Exhibited in Vaccine-Related Comments Across Several Canadian Cities

**Figure 3:**
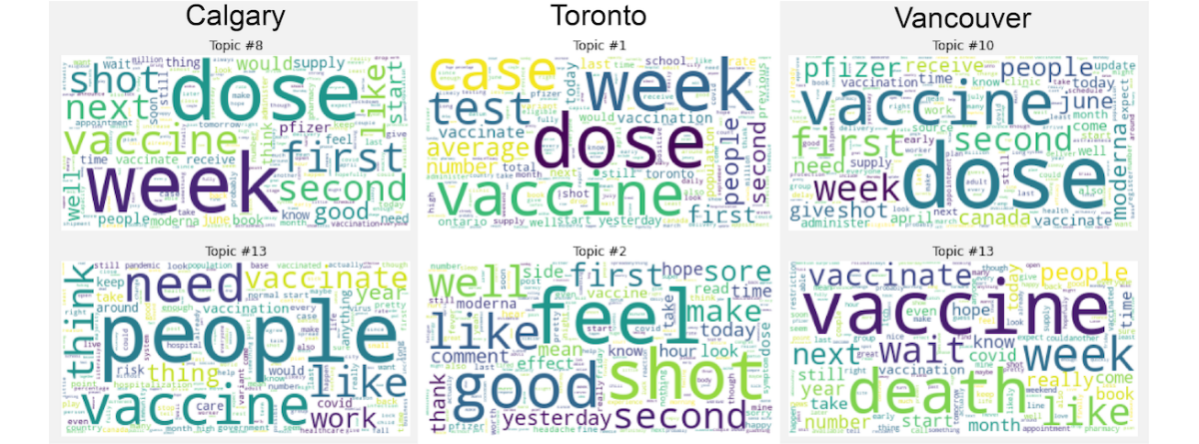
Word clouds for vaccine-related topics across the 3 cities.

To elucidate the effect of public sentiment on vaccination rates, 2 additional major Canadian cities, Toronto and Calgary, were analyzed using the same approach taken for Vancouver. A total of 13 distinct discussion topics were identified in r/toronto and 14 topics in r/calgary. Both had 2 dominant vaccine-related topics (Figure 3). In Toronto, these discussed vaccine uptake and postvaccine feelings. In r/calgary the 2 vaccine-related topics identified were vaccine uptake and concerns around vaccination rates. Top-scoring comments for each vaccine-related topic for Toronto and Calgary are shown in Multimedia Appendix 4.

Public sentiments for all emotions in Toronto and Calgary were significantly different between the 2 vaccine-related topics and between vaccine-related and non–vaccine-related comments (*P*<.001), except for the expression of fear in r/calgary comments (Multimedia Appendix 5). No significance was detected in the degree of fear between non–vaccine-related comments and those that discussed first and second dosages of COVID-19 vaccines.

In the Calgary Reddit community, comments discussing vaccination rates expressed lowest intensity for joy and the highest score for negative emotions (Multimedia Appendix 5). This coincides with Alberta having lower vaccination rates than British Columbia and Ontario (Multimedia Appendix 5). In Toronto, a higher degree of positive sentiment was observed in the vaccine-related comments (*P*<.001), with the highest median score occurring in comments that discussed vaccine side effects, followed by those pertaining to vaccine uptake (Multimedia Appendix 5).

#### Sentiments Toward Vaccine Uptake Significantly Differed Across Cities

**Figure 4:**
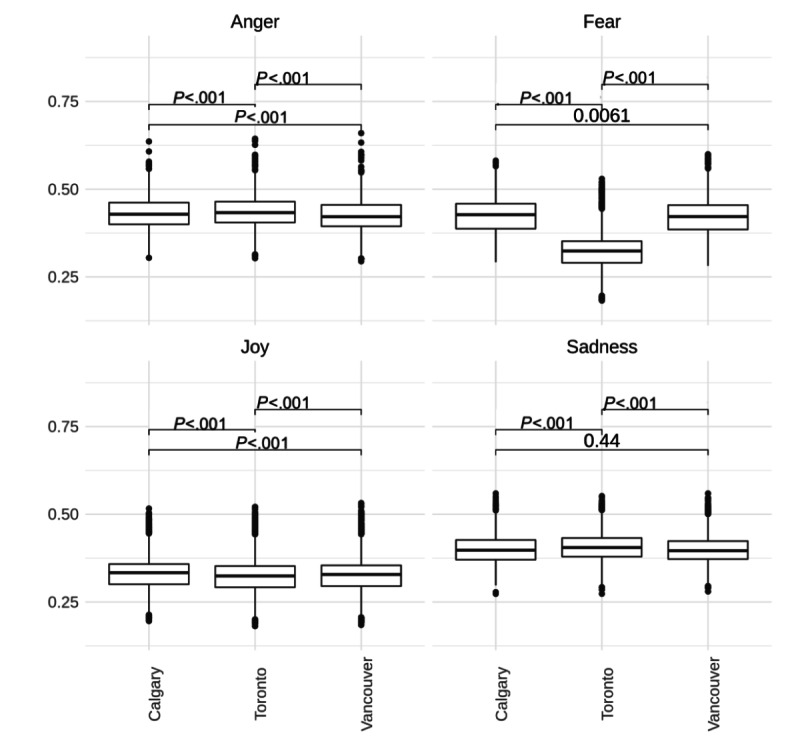
Box plots showing the distribution of emotional intensity scores for vaccine uptake. Comparisons between cities were made using the Mann–Whitney *U* test.

Sentiments toward vaccine uptake, the only vaccine-related topic shared by all 3 cities, differed significantly among cities across all emotions (*P*<.001; Figure 4). Toronto had the highest scores for anger and sadness and the lowest scores for joy and fear. Vancouver and Calgary had statistically the same scores for sadness, but Calgary had significantly higher scores for the other emotions.

## Discussion

### Principal Findings

In this study, we analyzed comments on the posts in r/vancouver, r/calgary, and r/toronto, which provide daily updates on case numbers, hospitalizations, and other COVID-19–related statistics. We found that the number of comments made on the posts from the Vancouver subreddit positively correlated with the number of new daily COVID-19 cases in British Columbia. From the comments, 13 topics were identified. Two topics were related to vaccines, 1 regarding vaccine uptake and the other about vaccine supply. The levels of discussion for both topics were linked to the total number of vaccines administered. Calgary and Toronto also discussed vaccine uptake, and sentiment scores for this topic differed across the 3 cities (*P*<.001).

Since July 2020, British Columbia has experienced fluctuations in COVID-19 cases and accompanying restrictions. Cases exponentially increased from 280 cases per day (7-day averages) at the beginning of November 2020 to 833 cases per day at the end of November 2020 [27]. In response, British Columbia issued a 2-week policy on November 7, 2020, prohibiting social gatherings outside of households [28]. This policy was renewed on November 19, 2020, in addition to the implementation of a mask mandate for public spaces [28].

Additionally, in April 2021, cases in British Columbia ranged from 873 to 1130 per day (7-day averages), forming a third peak. The increase in cases caused British Columbia to introduce travel restrictions for nonessential travel between British Columbia health authority boundaries, and to extend the state of emergency twice within the month of April 2021 [29-31].

These peaks in case numbers align with the 2 peaks in engagement on Reddit, and they were found to be significantly positively correlated. This trend suggests that when case numbers increase, more discussion is generated. This finding builds upon previous literature describing how people bond over the topic of COVID-19 on Twitter [32].

Furthermore, while the number of comments and the number of new cases of COVID-19 increase in tandem from October 2020, the number of comments peaks sooner and decreases at a faster rate than the number of new cases of COVID-19. This suggests that engagement on Reddit is a leading indicator of COVID-19 transmission, as is the case with Google searches, tweets, and Wikipedia page views [33].

However, for the second, higher peak in new cases of COVID-19 in April 2021, the number of Reddit comments increased, albeit at a lower rate, and did not exceed the peak in November 2020. Thus, rather than being a predictor of the number of cases, Reddit engagement patterns may instead be indicative of avoidance behaviors stemming from social media fatigue and the fear of COVID-19 arising from the stresses brought by the November wave [33].

To understand what people discuss on Reddit, we performed topic extraction and identified 13 topics through our model. Compared to studies analyzing themes circulating on Twitter at the beginning of the pandemic, in March 2020, we found that people have continued to compare case numbers, talk about restrictions, and share information on methods of preventing spread [34,35]. In addition, other topics that reflect British Columbia–specific policies and problems were discussed, such as the high transmission of COVID-19 within health care sectors such as long-term care homes during the second wave [36].

Two new, potentially more universal topics surrounding different facets of vaccines have also emerged. The first one addresses vaccine uptake, and the second one is about vaccine availability. Discussion for these topics began trending upward in November 2020, shortly after Pfizer and BioNTech announced the efficacy of their vaccine during phase 3 trials [37]. The level of discussion fluctuated as British Columbia prioritized vaccinating health care workers and residents of long-term care homes [38], but began to rise again in March 2021 as the public was administered vaccines [39].

At first, as the number of people vaccinated continued to grow, vaccine-related discussions increased as well. Comments on vaccine uptake have continued to increase, but have recently decreased for vaccine availability. Topic scores for vaccine availability were also generally higher than that of vaccine uptake until around March 2021. This is likely owing to reports of fluctuations and shortages in vaccine shipments [40,41]. Only recently has British Columbia been able to secure consistent and sufficient vaccine supplies enough to half the wait time between first and second doses [42,43]. Based on the significance of the Granger causality tests, it appears that discussion about either topic may have driven anticipation and demand for the vaccine.

Based on detailed discussions surrounding vaccines, we explored how people felt about them on Reddit by constructing a random forest regression model using tweets labeled with emotional intensity scores. The resulting RMSE values were considered acceptable, and the model was applied to predict emotional intensity scores for Reddit comments. Afterward, we extracted the cleaned comment with the highest score for each emotion. All 4 human authors agreed that the comments demonstrated the emotions they represented. To further validate the scores, a correlation matrix was created. As expected, the negative emotions (anger, fear, and sadness) were strongly positively correlated with each other but strongly negatively correlated with joy.

Observing the trends in emotional intensity of comments over time, we see that comments demonstrate more fear, anger, and sadness than joy [34]. However, comments expressing joy began increasing from April 2021, which coincides with the second peak in case numbers and rise in vaccinations. Since no similar changes were observed in November 2020, we concluded that vaccinations were related to the increase in comments expressing joy. To further confirm this, we compared emotional intensity scores between the 2 types of vaccine-related and non–vaccine-related comments and found that people expressed significantly more positive sentiments about vaccines.

Interestingly, the level of fear expressed about vaccine supply was not significantly different from that in non–vaccine-related comments, and both were significantly higher than vaccine uptake. Nonetheless, both vaccine topics had significantly lower anger and sadness scores than non–vaccine-related topics. This reflects concerns people have about not being able to get the vaccine [44].

Finally, we sought to compare vaccine sentiments across Canadian cities. In addition to discussing vaccine uptake as in Vancouver, Calgary, and Toronto, each had another regionally specific vaccine-related topics. For Toronto, these topics were about vaccine side effects. The word cloud had generally subjective and positive terms including “good” and “feel.” However, it’s emotional scores suggest that the topic may be polarized. Although it has the highest scores for joy, it also has the highest scores for fear. Nonetheless, both vaccine-related topics have significantly lower scores for anger and sadness than non–vaccine-related topics. This suggests that despite feeling generally positive about vaccines, there is still apprehension.

In Calgary, the second topic appears to be about vaccination rates. For this one, the sentiment is clear. The scores for negative emotions significantly exceed those for even non–vaccine-related topics. Since Calgary has the lowest vaccination rates and a lottery exclusively for vaccinated people as an incentive [45], it appears that Redditors are frustrated over the low uptake of vaccines.

When the common topic across all 3 cities—vaccine uptake—was compared, notable differences were observed. Calgary had the highest scores for fear, anger, and joy. Their highest-scoring comment for this topic embodies the first 2 emotions as it seems as though they had inadequate vaccine supply in the midst of high case numbers.

Interestingly, Toronto had the lowest scores for fear, despite also having the lowest scores for joy and the highest scores for sadness and anger. These sentiments could be attributed to frustration over booking vaccines [46] or having strict restrictions still in place despite high vaccination rates and decreasing case rates [47]. The low scores for fear than those for Calgary and Vancouver, however, are challenging to explain, especially since vaccine-related topics display higher fear than non–vaccine-related topics within Toronto’s own subreddit.

Based on our analyses, Reddit comments on posts in city-specific subreddits can be used to assess public sentiment toward COVID-19–related topics. Thus, it is a cost-effective and rapid way for officials to monitor citizens’ response to policies. Implementation of sentiment analysis to understand public perceptions of policies in Italy enhanced the accountability and responsiveness of policymakers [48]. Additionally, since engagement on Reddit correlated with COVID-19 cases and vaccination rates, discussion on social media can serve as predictors for real-world statistics.

Finally, our analyses were able to capture variations in sentiment about the same vaccine-related topic across the 3 cities. Accordingly, it is possible for officials to design policies that specifically target populations. For example, reassuring messaging about vaccine side effects and safety may be most useful for Toronto. In contrast, Vancouver could focus more on increasing vaccine supply and Calgary on appealing to vaccine-hesitant groups.

### Limitations

Despite the promise in our results, our analyses are not without limitations. First, the data were collected from subreddits, where anyone can comment. However, owing to the community-based nature of the subreddits, we assume that the comments are from commenters located in the cities we are studying. Therefore, the analysis is assumed to be specific to local and provincial policies.

Additionally, emotional intensity scores were assigned to Reddit comments based on tweets because no similarly labeled Reddit data set was available. Common libraries for sentiment analysis, including TextBlob and VADER, lacked specificity for COVID-19–related discussions. Since Twitter is similar to Reddit, in that people post and respond to short, publicly available messages under a username, we assumed that people would use similar language.

### Conclusions

Using comments on daily posts containing updates on COVID-19 statistics from a location-specific subreddit, we were able to relate changes in web-based engagement, discussion, and emotional expression to case counts and vaccination rates. Topics relevant to local news and policies were identifiable, as were attitudes toward measures to curb disease spread, such as vaccines. Overall, our study shows that data from social media can be used to better understand concerns and sentiments surrounding the pandemic at the local level, which enables more targeted and publicly acceptable policies.

## Appendix

Multimedia Appendix 1 Line plot comparing Jaccard similarity (red) and coherence (blue) metrics for LDA models created with 1 to 15 topics. The black vertical line denotes the optimal number of topics.

Multimedia Appendix 2 Word clouds for topics extracted from r/vancouver.

Multimedia Appendix 3 Sentiment analysis in r/vancouver.

Multimedia Appendix 4 Examples of highest-scoring comments for vaccine-related topics for Toronto and Calgary. Approximately the first 25 words of each comment are shown for conciseness.

Multimedia Appendix 5 Comparison of sentiment scores for vaccine-related topics in Calgary and Toronto.

## Abbreviations

IDF: inverse document frequency
LDA: Latent Dirichlet Allocation
NLTK: Natural Language Toolkit
PRAW: Python Reddit API Wrapper
RCT: randomized controlled trial
RMSE: root mean square error
TF: term frequency
TF–IDF: term frequency–inverse document frequency
