# Supplementary material for: Comparing Public Sentiment Toward COVID-19 Vaccines Across Canadian Cities: Analysis of Comments on Reddit
Source: J Med Internet Res. 2021 Sep 24;23(9):e32685. doi: 10.2196/32685 (PMC8477909; doi:10.2196/32685)

Multimedia Appendix 1: Line plot comparing Jaccard similarity (red) and coherence (blue) metrics for LDA models created with 1 to 15 topics. The black vertical line denotes the optimal number of topics.
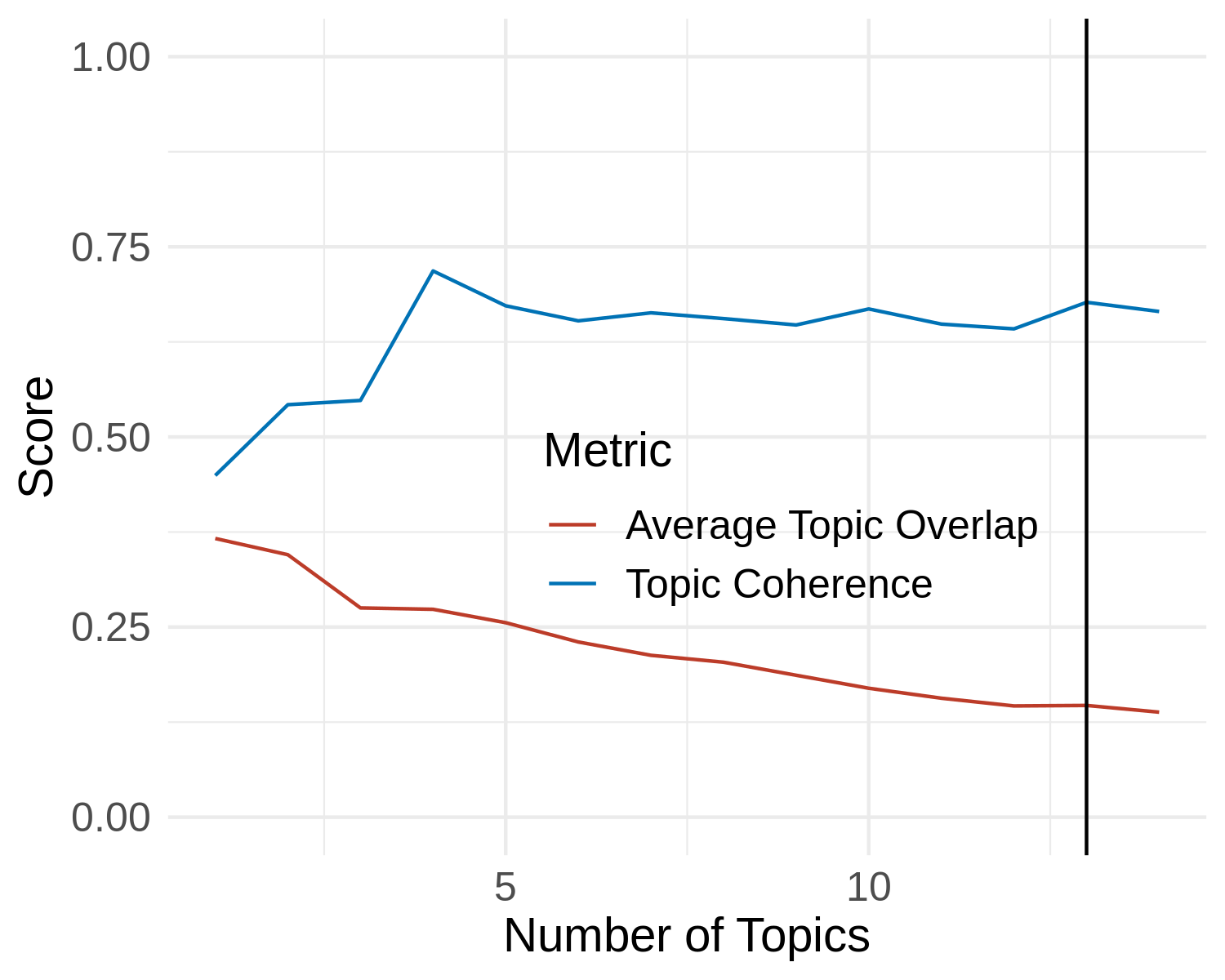

Supplement: Multimedia Appendix 1 [file jmir_v23i9e32685_app1.docx]
