# Supplementary figures and images for: Comparing Public Sentiment Toward COVID-19 Vaccines Across Canadian Cities: Analysis of Comments on Reddit
Source: J Med Internet Res. 2021 Sep 24;23(9):e32685. doi: 10.2196/32685 (PMC8477909; doi:10.2196/32685)

Multimedia Appendix 2: Word clouds for topics extracted from r/vancouver


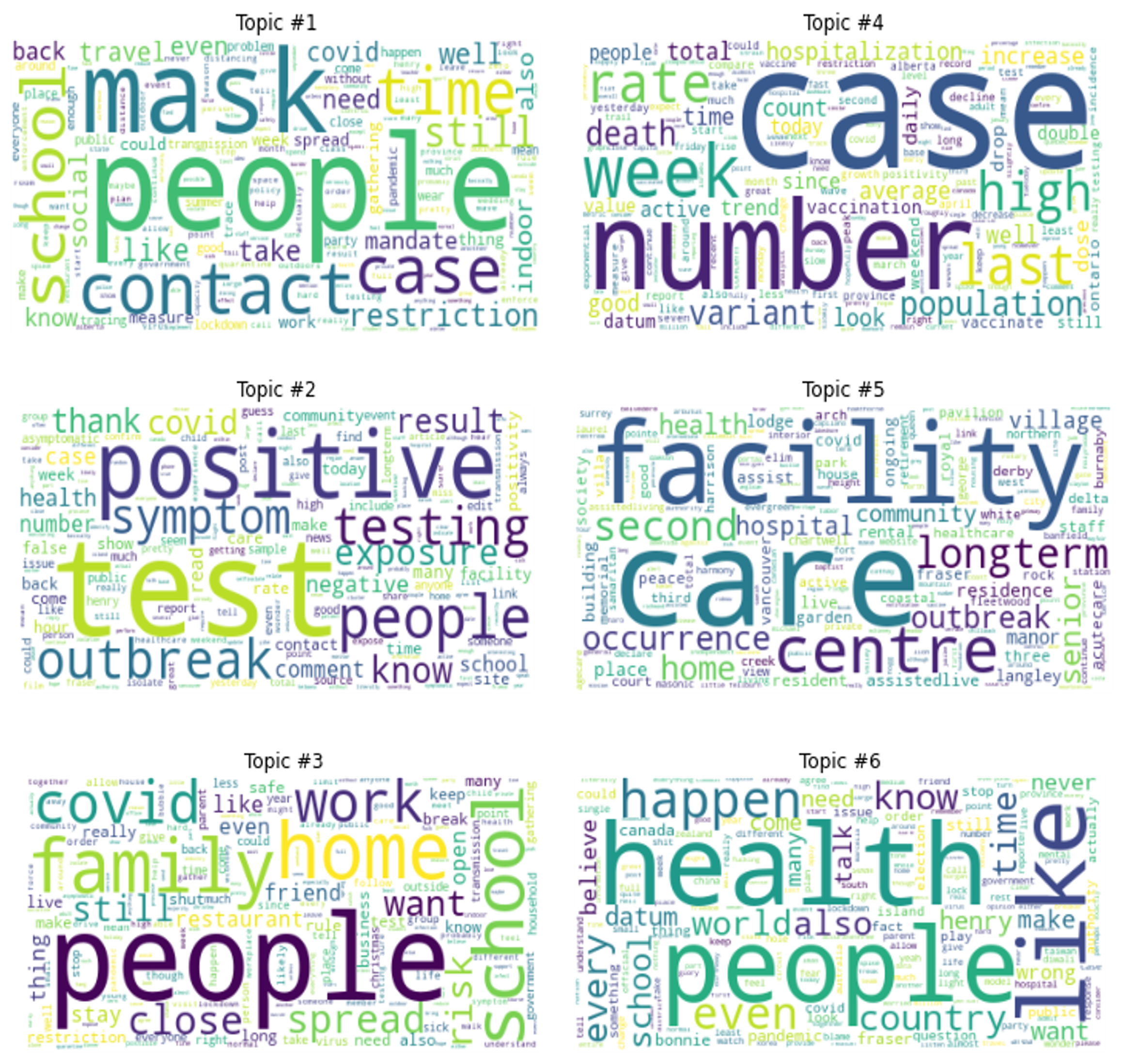


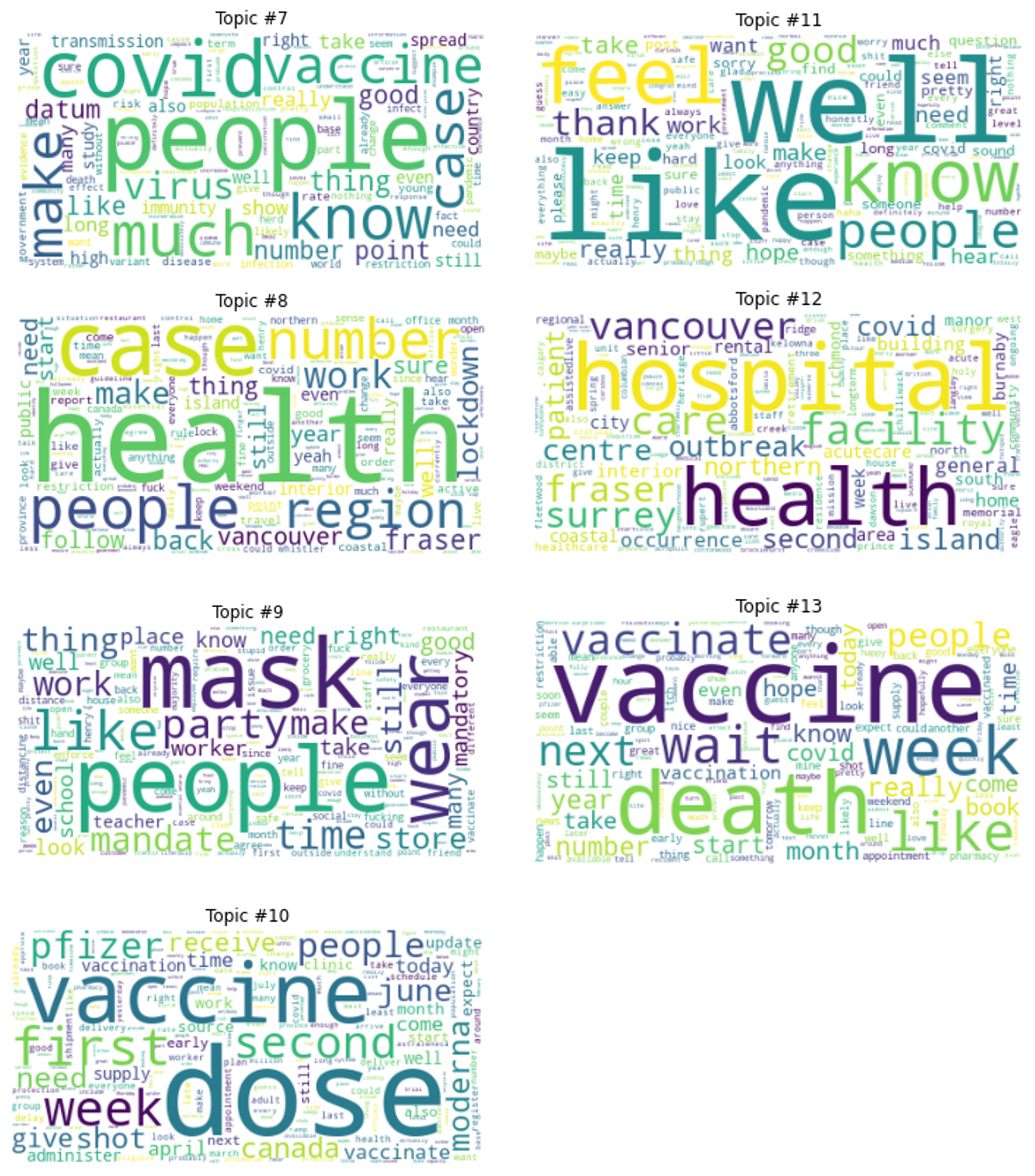

Supplement: Multimedia Appendix 2 [file jmir_v23i9e32685_app2.docx]
