# Supplementary material for: Comparing Public Sentiment Toward COVID-19 Vaccines Across Canadian Cities: Analysis of Comments on Reddit
Source: J Med Internet Res. 2021 Sep 24;23(9):e32685. doi: 10.2196/32685 (PMC8477909; doi:10.2196/32685)
Supplement: Multimedia Appendix 3 [file jmir_v23i9e32685_app3.docx]

Multimedia Appendix 3: Sentiment analysis in r/vancouver

Table 1. RMSE values from random forest regression models trained on tweets to predict emotional intensity scores.

| Emotion | RMSE |
| --- | --- |
| Joy | 0.083 |
| Sadness | 0.068 |
| Anger | 0.074 |
| Fear | 0.079 |


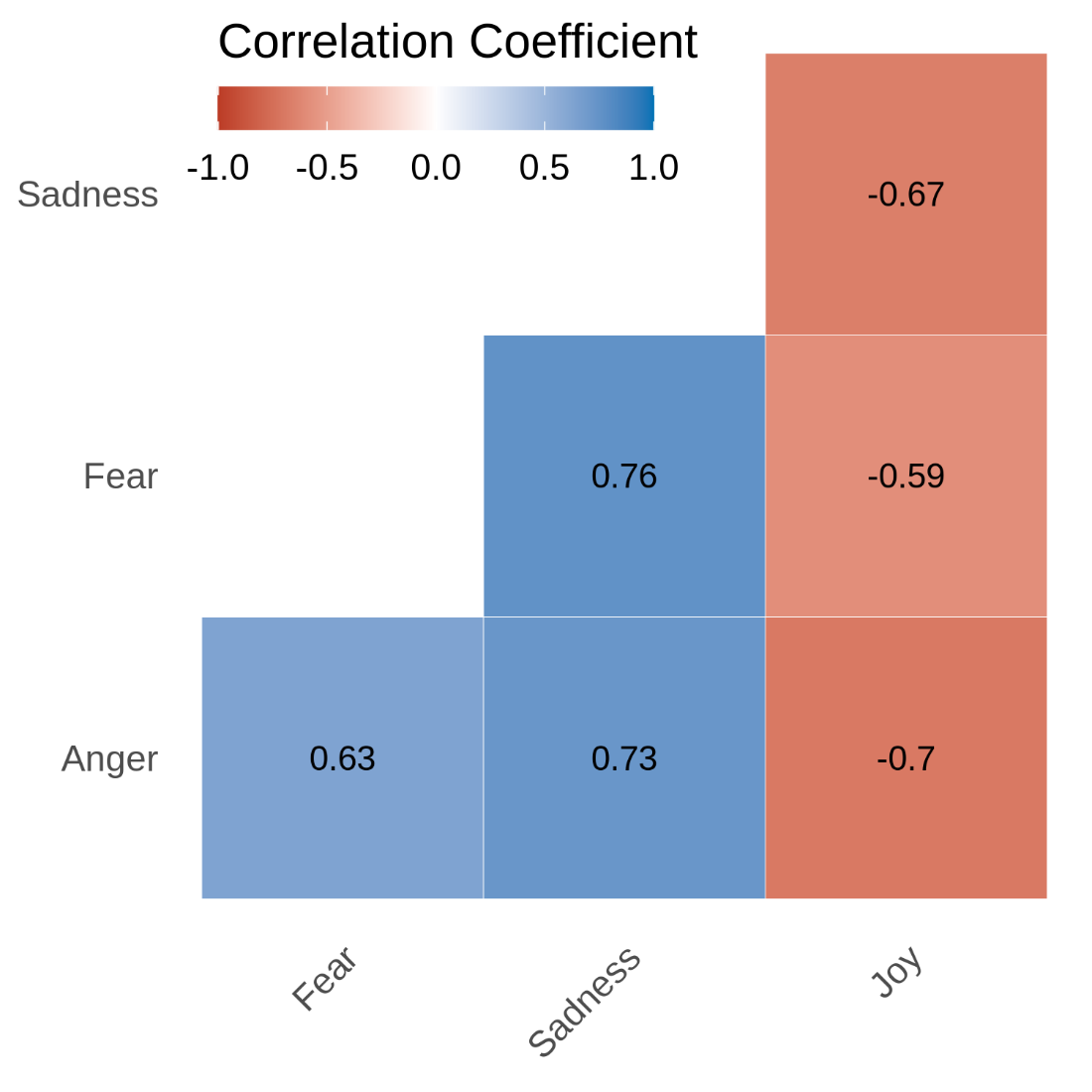


Figure 1. Heatmap showing the correlation coefficient between emotional intensity scores.


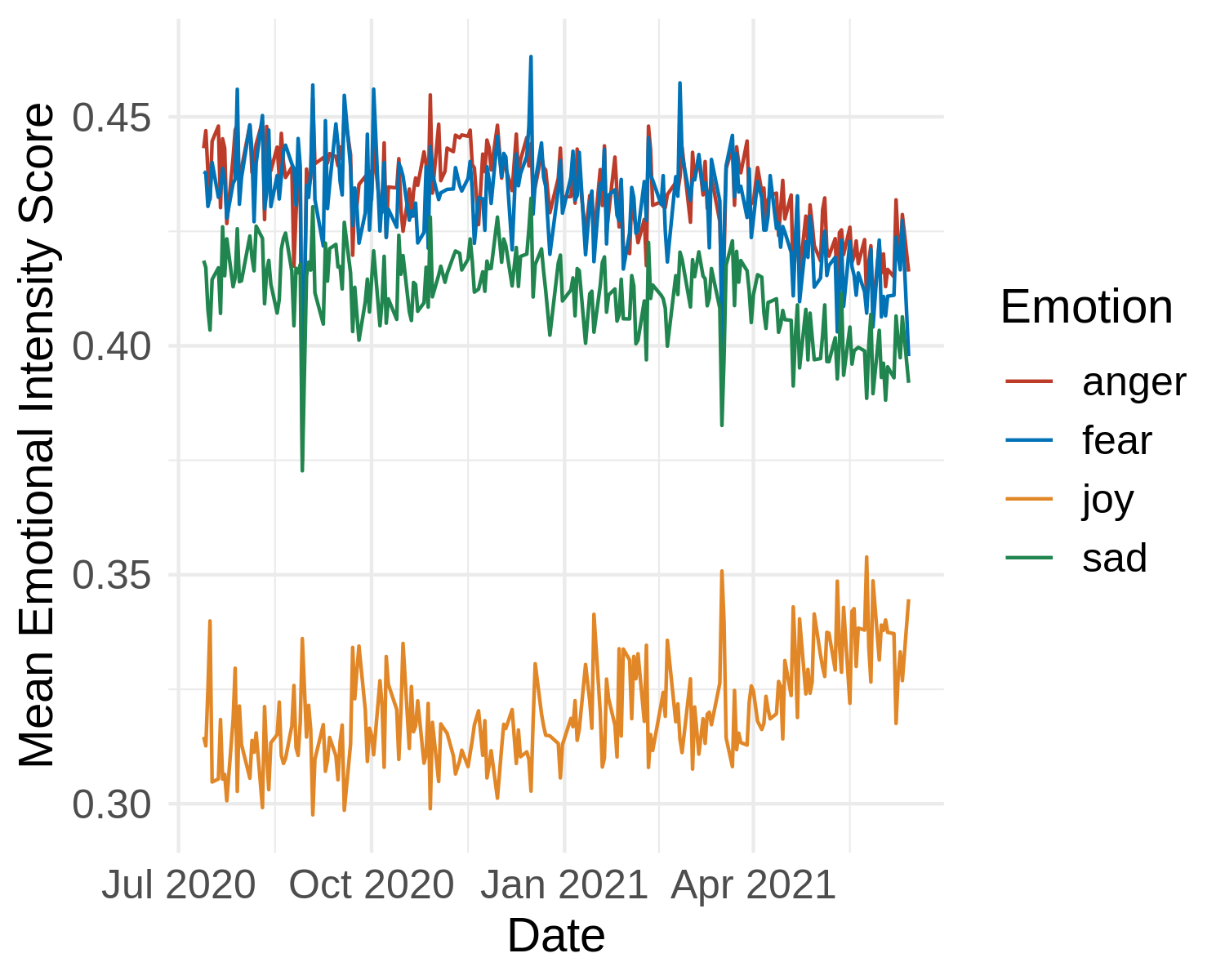


Figure 2. Line plot displaying the daily average emotional intensity score split by emotion.
