# Supplementary material for: Comparing Public Sentiment Toward COVID-19 Vaccines Across Canadian Cities: Analysis of Comments on Reddit
Source: J Med Internet Res. 2021 Sep 24;23(9):e32685. doi: 10.2196/32685 (PMC8477909; doi:10.2196/32685)
Supplement: Multimedia Appendix 4 [file jmir_v23i9e32685_app4.docx]

Multimedia Appendix 4. Examples of highest-scoring comments for vaccine-related topics for Toronto and Calgary. Approximately the first 25 words of each comment are shown for conciseness.

| City | Topic | Clean Comment |
| --- | --- | --- |
| Toronto | 1 | depend situation go usa could still well idea vaccination even though suppose get sort vaccine delivery summer go vaccinate state not care residency citizenship requirement |
| Toronto | 2 | think little bit increase heart rate second one day along incredibly sore arm bit flush face temperature rise swollen neck lymph node feeling shortness breath |
| Calgary | 8 | way low vaccine freezer coming weekly need close see consistently high number soon suggest e4veryone start call email mla daily ask alberta government make vaccination priority |
| Calgary | 13 | early today concern shift ill able get vaccination adult concern year old child approve vaccination available seem level masking cohort no socializing likely require group also majority vaccinate an |
