# Supplementary material for: Comparing Public Sentiment Toward COVID-19 Vaccines Across Canadian Cities: Analysis of Comments on Reddit
Source: J Med Internet Res. 2021 Sep 24;23(9):e32685. doi: 10.2196/32685 (PMC8477909; doi:10.2196/32685)
Supplement: Multimedia Appendix 5 [file jmir_v23i9e32685_app5.docx]

Multimedia Appendix 5: Comparison of sentiment scores for vaccine-related topics in Calgary and Toronto


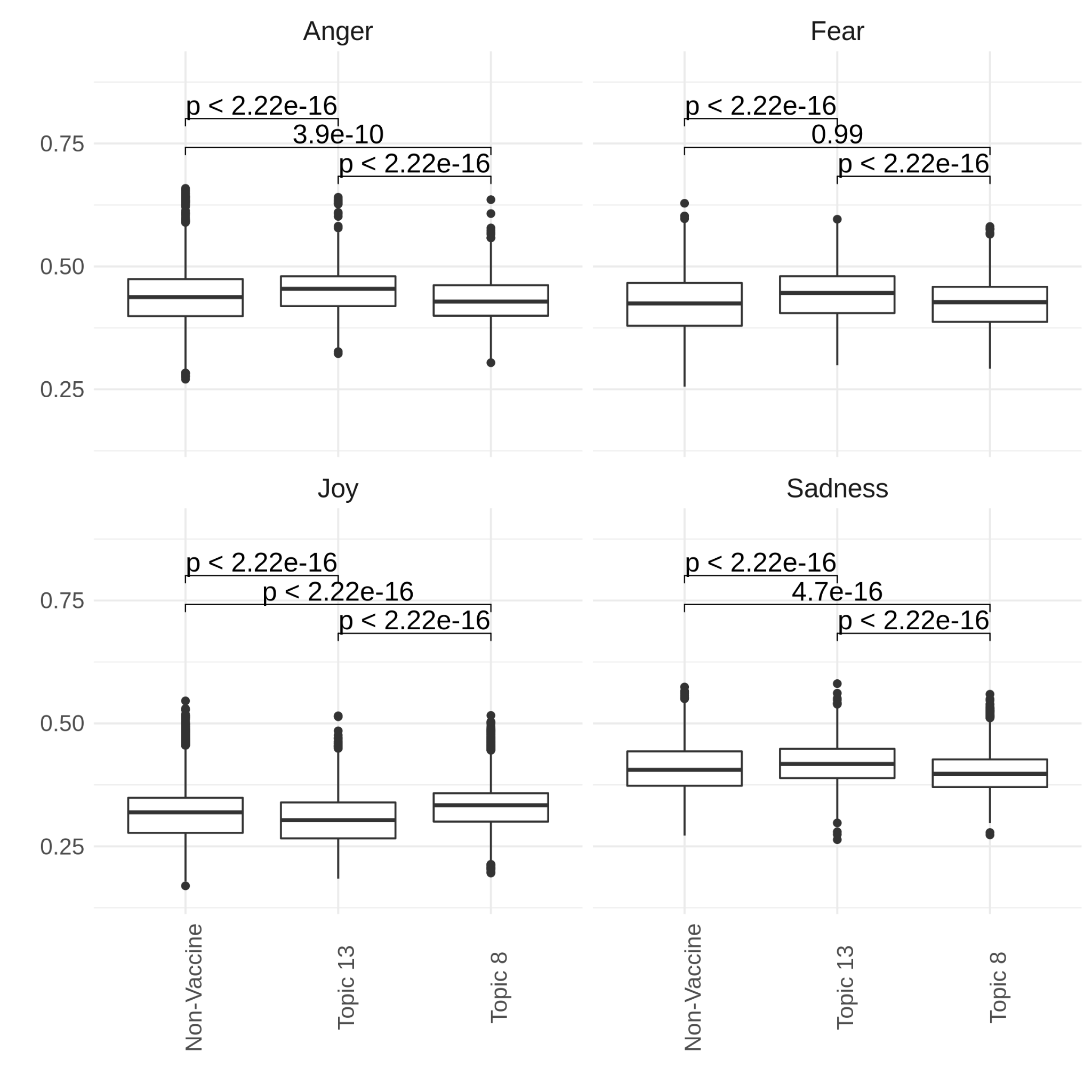


Figure 1. Box plots showing the distribution of emotional intensity scores for Calgary. Comparisons between groups were made using the Mann-Whitney U test.


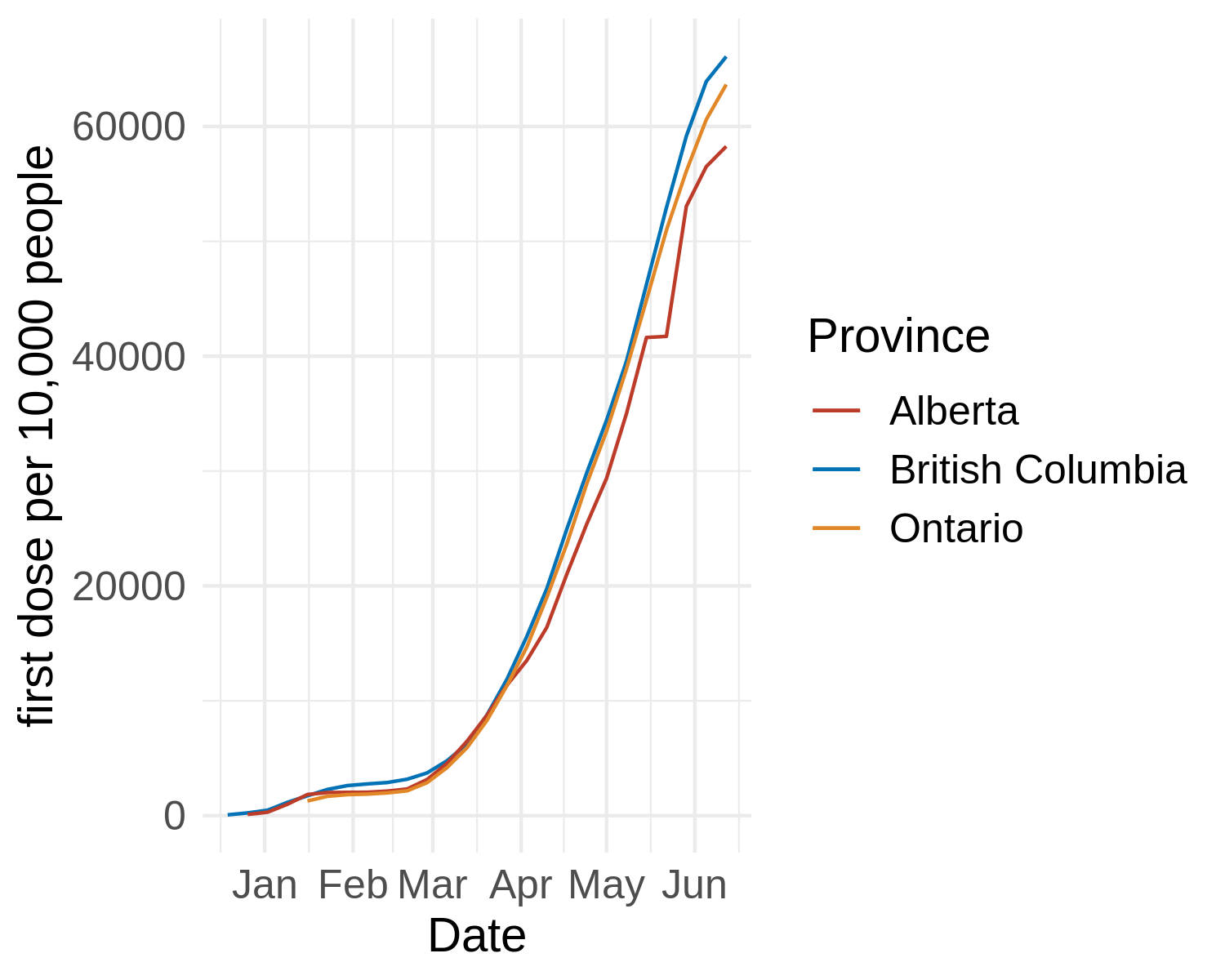


Figure 2. Line plots showing the per capita number of vaccines given over time for the three cities.


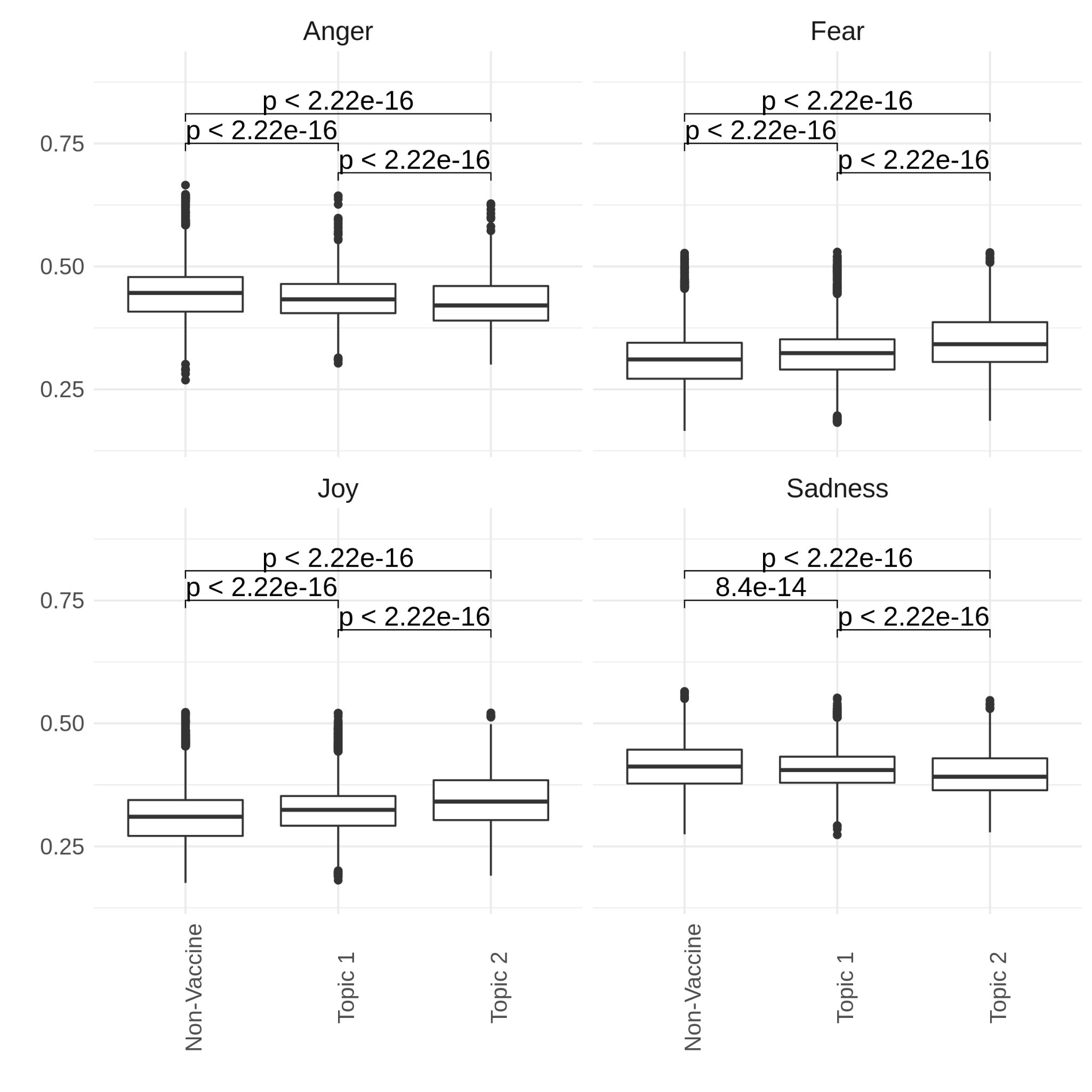


Figure 3. Box plots showing the distribution of emotional intensity scores for Toronto. Comparisons between groups were made using the Mann-Whitney U test.
